# Association of VCP/p97 Expression with Clinicopathological Features and Overall Survival in Endometrioid Endometrial Carcinoma

**DOI:** 10.3390/biomedicines14102248

**Published:** 2026-10-04

**Authors:** Akgül Arıcı, Elif Akçay, Kübra Arslan, Büşra Demir, Reşit Doğan Köseoğlu

**Affiliations:** 1 Department of Medical Pathology, School of Medicine, Tokat Gaziosmanpaşa University, Tokat 60250, Türkiye; drelf186@windowslive.com (E.A.); rdogankoseoglu@gmail.com (R.D.K.); 2 Department of Medical Pathology, Gaziantep City Hospital, Gaziantep 27470, Türkiye; kbrab91@gmail.com; 3 Department of Medical Pathology, School of Medicine, Harran University, Şanlıurfa 63300, Türkiye; busrademir@harran.edu.tr

**Keywords:** endometrial cancer, endometrioid endometrial carcinoma, valosin-containing protein, immunohistochemistry, tumor progression

## Abstract

**Background and Aim**: Endometrial cancer is considered one of the most common cancers of the female genital system, with endometrioid endometrial carcinoma (EEC) being the most common subtype. Valosin-containing protein (VCP), or p97, acts as an important regulator of cellular protein homeostasis and is thought to play a role in the progression and prognosis of various tumors. This study aimed to evaluate the association of immunohistochemical VCP/p97 expression with clinicopathological features and overall survival in patients with EEC. **Methods**: This retrospective single-center study utilized hysterectomy specimens obtained from 85 patients diagnosed with EEC between January of 2010 and December of 2022. All cases were retrospectively restaged according to the 2023 FIGO staging system. VCP/p97 expression was evaluated immunohistochemically using a semi-quantitative scoring system based on staining intensity and the percentage of positive tumor cells. Patients with EEC were divided into groups with low and high VCP/p97 expression. Clinicopathological associations, alternative cut-off sensitivity analyses, Kaplan–Meier survival analysis, and Cox regression analyses were performed. **Results**: Low VCP/p97 expression was detected in 49 cases (57.6%), whereas high expression was detected in 36 cases (42.4%). High VCP/p97 expression was significantly associated with older age, larger tumor size, high FIGO grade, deep myometrial invasion, lymphovascular invasion, cervical and adnexal involvement, pathological lymph node metastasis, distant metastasis, and advanced FIGO stage. Overall survival was significantly worse in the high-expression group (log-rank *p* < 0.001). In the multivariate Cox regression analysis, high VCP/p97 expression remained associated with poorer overall survival. **Conclusions**: High immunohistochemical VCP/p97 expression is associated with an adverse clinicopathological phenotype and poorer overall survival in EEC. VCP/p97 may be a considered a candidate tissue-based marker associated with tumor progression features; however, its independent prognostic utility needs to be validated in larger independent cohorts.

## 1. Introduction

Endometrial cancer is one of the most common malignancies of the female genital system, particularly in developed and upper-middle-income countries. It is the sixth most common cancer among women worldwide and is predominantly observed in postmenopausal women, with a mean age at diagnosis of 63 years [1]. The lifetime risk of developing endometrial cancer in women has been reported to be approximately 3% [2]. Endometrioid endometrial carcinoma (EEC) is the most common histological subtype of endometrial cancer. Although endometrioid-type tumors are generally considered estrogen-related, low-grade tumors diagnosed at an early stage, there is marked heterogeneity in the clinical course within this group. While some low-grade and uterus-confined cases show favorable long-term prognosis, other cases with a similar histological type may have a poor prognostic course [3,4].

Risk stratification and treatment planning for patients with endometrial cancer are traditionally based on the surgical staging system defined by the International Federation of Gynecology and Obstetrics (FIGO) [3,5]. Conventional clinicopathological parameters such as FIGO stage, histopathological tumor grade, depth of myometrial invasion, lymphovascular invasion, and lymph node involvement status are among the factors that play important roles in predicting patient prognosis and shaping adjuvant treatment decisions [6]. In recent years, molecular classification has begun to be incorporated into current guidelines for endometrial cancer risk stratification. The POLE-mutant, mismatch repair (MMR)-deficient, p53-abnormal, and no specific molecular profile subgroups defined by The Cancer Genome Atlas (TCGA) provide a framework that reflects the biological diversity of endometrial cancer [1,7,8]. However, the high cost of molecular analyses, their requirement for advanced technological infrastructure, and their frequent inaccessibility in routine pathology laboratories drive the need for new tissue-based biomarkers that can be assessed using more economical, reproducible, and routine immunohistochemical methods [9,10]; in particular, the survival differences observed among patients with the same stage and grade indicate the importance of finding independent prognostic markers that more sensitively reflect the biological behavior of the tumor [2].

Valosin-containing protein, also known as Valosin-containing protein/p97 (VCP/p97), is a highly conserved and multifunctional homohexameric protein complex belonging to the AAA+ ATPase family which plays a central role in the maintenance of cellular protein homeostasis. VCP/p97 uses the energy derived from ATP hydrolysis to extract ubiquitinated proteins from membranes, protein complexes, or chromatin structures and directs these substrates to proteasomal degradation, recycling, refolding, or other cellular pathways. As such, it coordinates cell survival under metabolic and environmental stress conditions [11,12]. VCP/p97 has been shown to play a role in numerous fundamental cellular processes, such as endoplasmic reticulum-associated protein degradation (ERAD), the ubiquitin–proteasome system, autophagy, aggresome clearance, mitophagy, DNA damage response, cell cycle regulation, and apoptosis. This broad functional network suggests that VCP/p97 may be a critical molecule not only for normal cellular homeostasis, but also for cancer cells with high proliferative capacity and increased proteotoxic stress [13,14,15,16,17].

Cancer cells may become more dependent on protein quality control systems than normal cells due to their rapid proliferation, aneuploidy, genomic instability, increased protein synthesis, and hypoxic/metabolic stress. The ubiquitin–proteasome system is of central importance in cancer biology, as it removes misfolded or damaged proteins, regulates cell cycle proteins, and controls tumor suppressor and oncogenic signaling pathways [15]. Since VCP/p97 functions in critical sorting and substrate extraction steps upstream of the proteasome in this system, it may provide an important adaptation mechanism through which tumor cells can better tolerate proteotoxic stress [11]. Therefore, overexpression of VCP/p97 may contribute to biological processes associated with proliferation, invasion, metastasis, escape from apoptosis, and treatment resistance in cancer cells [14]. In EEC, these VCP/p97-related mechanisms may be relevant to pathological features reflecting local invasion and tumor dissemination. By supporting proteostasis and cellular stress adaptation, increased VCP/p97 expression may help tumor cells to survive under the hypoxic, metabolic, and proteotoxic stress conditions commonly encountered during tumor growth. In addition, experimental studies have suggested that VCP/p97 may influence the epithelial–mesenchymal transition, cytoskeletal organization, and tumor cell motility [18,19]. These mechanisms may provide a biological rationale for investigating whether VCP/p97 expression is associated with pathological features such as deep myometrial invasion, lymphovascular invasion, lymph node metastasis, distant metastasis, and advanced FIGO stage in EEC; however, these potential relationships remain inferential and require clinicopathological validation in human tumor tissue.

The prognostic value of VCP/p97 in cancers has been investigated in numerous clinical studies involving different organ systems. Studies in the literature have shown that increased VCP/p97 expression is associated with tumor progression, high histological grade, lymph node metastasis, distant organ metastasis, and poor overall survival in many solid and hematological malignancies, including pancreatic endocrine neoplasms, colorectal carcinoma, gastric cancer, hepatocellular carcinoma, non-small cell lung cancer, breast cancer, prostate cancer, follicular thyroid carcinoma, gingival cancer, glioma, ovarian cancer, choriocarcinoma, and acute myeloid leukemia [20,21,22,23,24,25,26,27,28,29,30,31,32,33,34].

Data related to VCP/p97 in gynecological malignancies are limited. In vitro studies on endometrial tissue and related neoplasms suggest that ubiquitin-dependent protein degradation pathways and signal transduction mechanisms may play important roles in the regulation of tumor biology [35]. In particular, it has been demonstrated that inhibition of VCP/p97 or blockade of this pathway with agents such as disulfiram triggers proteotoxic stress, endoplasmic reticulum stress, and apoptosis in endometrial cancer cell lines [36].

Although these laboratory data indicate that VCP/p97 may play an important role in maintaining the viability of endometrial cancer cells, the immunohistochemical expression profile of this protein in human EEC tissues, its relationship with clinicopathological parameters, and its association with the long-term survival outcomes of patients have not yet been sufficiently investigated at the clinical level. Despite the strong clinical value of classical prognostic parameters in the context of endometrial cancer, the observation of different clinical courses within the same histological type and stage necessitates the investigation of new biological markers.

This study aimed to evaluate the associations between immunohistochemical VCP/p97 expression, clinicopathological features, and overall survival in patients with EEC.

## 2. Materials and Methods

### 2.1. Patients

This study was planned as a single-center study with a retrospective design. A total of 126 patients diagnosed with EEC between January of 2010 and December of 2022 were initially identified via hysterectomy materials from the archives of Tokat Gaziosmanpaşa University Faculty of Medicine, Department of Pathology. As pathological lymph node status was one of the main clinicopathological variables evaluated in this study, only patients who underwent surgical lymph node assessment and had pathologically evaluable nodal status were included. Accordingly, 25 patients who did not undergo surgical lymph node evaluation/dissection were excluded to avoid classifying clinically unevaluated nodal status as pathologically node-negative. In addition, 9 patients with missing clinicopathological or survival information, 5 patients whose archival material could not be accessed, and 2 patients whose paraffin blocks did not contain sufficient tumor tissue for immunohistochemical evaluation were excluded. After these exclusions, the final study cohort consisted of 85 patients.

Clinicopathological parameters, including age, tumor diameter, FIGO grade, depth of myometrial invasion, lymphovascular invasion, cervical invasion, adnexal involvement, lymph node metastasis, distant metastasis, FIGO stage, and survival status, were retrospectively obtained from pathology reports and hospital electronic system records. All cases were retrospectively restaged according to the 2023 FIGO staging system based on the available pathology report data, in order to implement a common staging framework. Myometrial invasion was categorized into two groups—namely, <50% and ≥50%—based on the depth of tumor invasion into the myometrium. As information on focal versus diffuse lymphovascular invasion was not consistently available in the pathology reports, lymphovascular invasion was recorded as either present or absent. Cervical invasion, adnexal involvement, lymph node metastasis, and distant metastasis were recorded as absent or present. FIGO grade was evaluated as grade I, II, or III; for statistical analyses, cases were grouped as grade I–II and grade III. FIGO stage was classified as stage I–II and stage III–IV for comparisons between early- and advanced-stage disease.

Overall survival was defined as the time from surgery to death from any cause or to the date of the last data collection; patients alive at the last follow-up date were censored. The last data collection date was determined as June of 2025. Survival status and dates of death were verified using official national population records integrated into the hospital system. No case was lost to follow-up in terms of survival status. Data regarding recurrence status, disease-free survival, adjuvant treatment modalities, comorbidities, and cause-specific mortality were not available, and therefore were not included in the analyses.

The study was conducted in accordance with the Declaration of Helsinki and was approved by the Clinical Research Ethics Committee of the Faculty of Medicine, Tokat Gaziosmanpaşa University (Approval No. 24-KAEK-063).

### 2.2. Immunohistochemistry

Hematoxylin and eosin-stained slides from all cases were re-evaluated to confirm the diagnosis and select appropriate tumor areas. For each case, a formalin-fixed, paraffin-embedded tissue block that adequately represented the tumor, did not contain necrosis or artifacts, and was considered suitable for immunohistochemical analysis was selected. For immunohistochemical analysis, 4 µm thick sections were prepared from formalin-fixed, paraffin-embedded tissue blocks and mounted on positively charged slides. Immunohistochemical staining was performed using a Leica Bond-Max fully automated immunohistochemistry staining system (Leica Biosystems, Nussloch, Germany). VCP/p97 expression was evaluated using a primary antibody against VCP/p97 (ab11433, 1:500; Abcam, Cambridge, UK). Antigen retrieval was performed using Bond Epitope Retrieval Solution 1 (Leica Biosystems), according to the validated laboratory protocol for this antibody. Immunohistochemical detection was carried out using the Bond Polymer Refine Detection Kit (Leica Biosystems). Chromogenic visualization and hematoxylin counterstaining were performed according to the manufacturer’s automated staining protocol.

**Figure 1:**
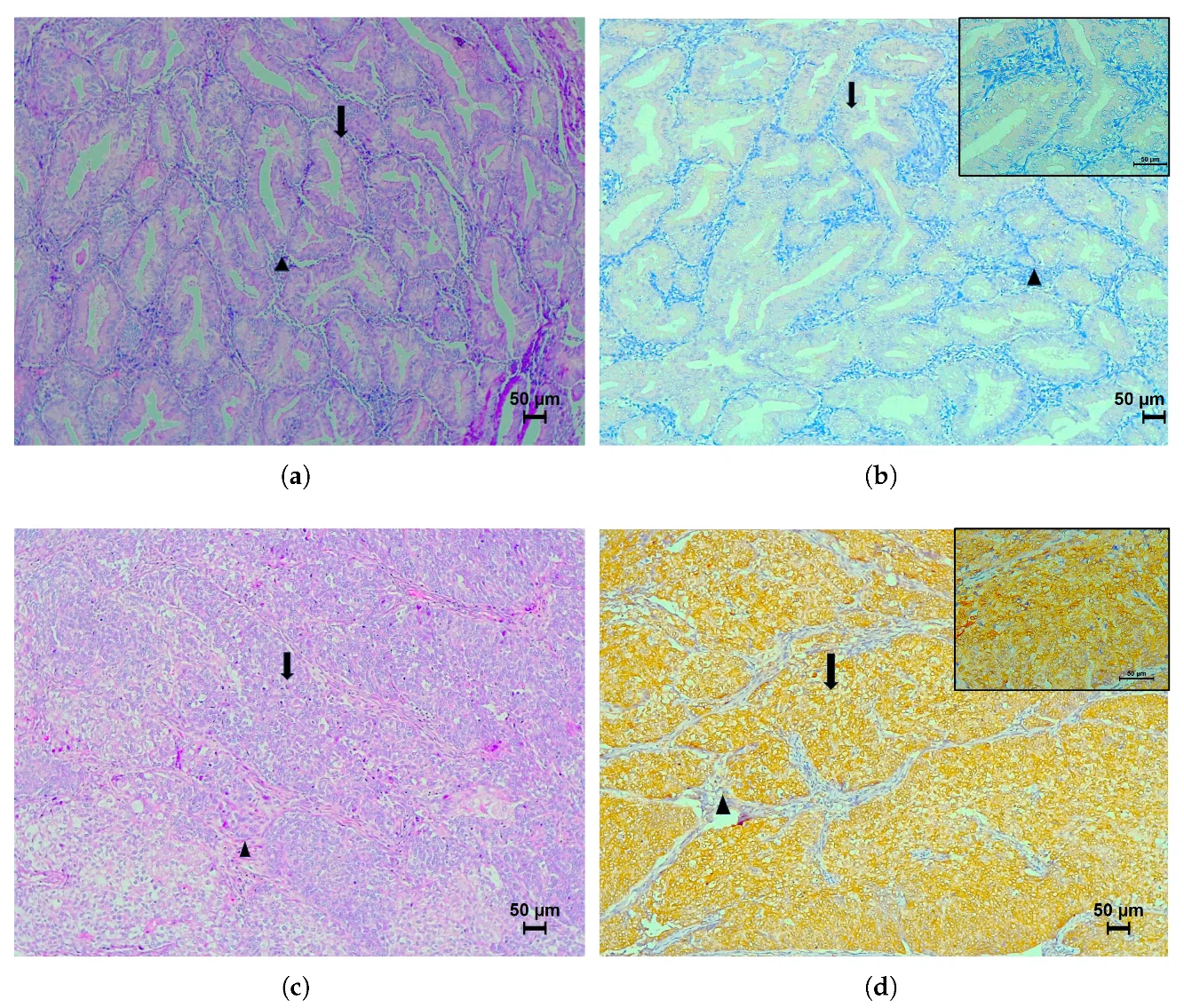
Representative histopathological and immunohistochemical images of low and high valosin-containing protein/p97 (VCP/p97) expression in endometrioid endometrial carcinoma. (**a**,**b**) Low VCP/p97 expression showing weak cytoplasmic staining in tumor cells. (**c**,**d**) High VCP/p97 expression showing strong cytoplasmic staining in tumor cells. (**a**,**c**) Hematoxylin and eosin staining; (**b**,**d**) VCP/p97 immunohistochemical staining. Tumor glands are marked with arrows, whereas the surrounding stromal areas are marked with triangles. Insets in (**b**,**d**) show higher-magnification views of cytoplasmic VCP/p97 staining in tumor cells. Original magnification, 100×; insets, 400×. Scale bars: 50 µm. The images are presented without post-acquisition color balance adjustment.

During immunohistochemical evaluation, cytoplasmic staining in tumor cells was considered positive for VCP/p97 expression, while nuclear staining (if present) was not included in the scoring procedure. Kidney tissue served as the positive control for VCP/p97 immunostaining and was evaluated in parallel with the study slides to confirm staining adequacy; a separate negative control slide was not used. Staining evaluation was performed by two independent researchers who were blinded to the clinicopathological and survival data of the cases. VCP/p97 staining was evaluated in ten randomly selected high-power fields on each section, and both the staining intensity and the percentage of positive tumor cells were taken into account during the evaluation. Staining intensity was organized into four categories via scoring; in particular, cases with no staining were scored as 0, cases with weak staining as 1, cases with moderate staining as 2, and cases with strong staining as 3. The percentage of positive tumor cells was also scored, as follows: cases with no positive tumor cells were scored as 0, cases with <10% positive tumor cells as 1, cases with 10–50% positive tumor cells as 2, and cases with >50% positive tumor cells as 3. The final VCP/p97 expression score was calculated by multiplying the staining intensity score by the reactivity score based on the percentage of positive tumor cells [27]. As no universally accepted immunohistochemical cut-off value has been established for VCP/p97 expression in EEC, candidate final score thresholds of >2, >3, >4, and >6 were considered and evaluated through sensitivity analyses. For the main analysis, a final score of >4 was used to define high VCP/p97 expression because this threshold provided a more balanced group distribution and an estimable Cox regression model, when compared with the other explored thresholds. Accordingly, cases with an expression score of ≤4 were classified as having low VCP/p97 expression, whereas cases with a score of >4 were classified as having high VCP/p97 expression (Figure 1). The use of this cut-off is considered exploratory, and is further addressed in the sensitivity analyses. In cases with discrepancies between the two evaluators, the slides were jointly re-evaluated and the final score was determined by consensus. For tumors with heterogeneous staining, the predominant staining pattern was used for scoring.

### 2.3. Statistical Analysis

Statistical analyses were performed using IBM SPSS Statistics for Windows, Version 27.0 (IBM Corp., Armonk, NY, USA). Continuous variables were expressed as mean ± standard deviation or median (range), as appropriate, and categorical variables as number (%). VCP/p97 expression was analyzed in two groups; namely, low and high expression. The normality of continuous variables was assessed using visual and analytical methods. Depending on the distribution characteristics, Student’s *t*-test or the Mann–Whitney U test was used to compare continuous variables between two groups. Categorical variables were compared using the chi-square test or Fisher’s exact test, as appropriate.

Overall survival was calculated, in months, from the date of surgery to the date of death or the date of last data collection. Patients who were alive at the last data collection date were censored. Overall survival was estimated using the Kaplan–Meier method, with between-group differences assessed using the log-rank test. Numbers at risk were displayed below the survival curves at predefined time points.

Cox proportional hazards regression analysis was carried out to identify factors associated with overall survival. In the first step, univariate Cox regression analysis was performed for each clinicopathological variable. Next, variables that were considered clinically relevant and those found to be associated with survival in the univariate analysis were evaluated for inclusion in the multivariate model. Considering the limited number of events, the multivariate Cox regression model was restricted to VCP/p97 expression, age, FIGO grade, and FIGO stage in order to reduce the risk of model overfitting. Lymph node metastasis was not included in the multivariate model as it is incorporated into FIGO stage and is closely related to advanced-stage disease. The results are presented as hazard ratios (HRs), 95% confidence intervals (CIs), and *p*-values.

The proportional hazards assumption for the Cox regression model was assessed using Schoenfeld residual-based tests. Both variable-specific and global tests were evaluated. A non-significant test result was interpreted as showing no clear evidence against the proportional hazards assumption.

Cut-off sensitivity analyses were performed to evaluate the robustness of the VCP/p97 expression threshold. Alternative final score thresholds of >2, >3, >4, and >6 were used to dichotomize VCP/p97 expression. For each threshold, the number of cases, number of events, log-rank *p*-value, and univariate Cox regression result were evaluated. When sparse events, complete separation, or a very small subgroup size precluded reliable hazard ratio estimation, the hazard ratio was reported as not estimable. As the cut-off was evaluated retrospectively, these analyses were considered exploratory. Interobserver agreement for VCP/p97 immunohistochemical scoring was assessed using the independent observer scores before consensus review. Unweighted Cohen’s kappa was calculated for the dichotomized low/high VCP/p97 expression classification. Linear weighted kappa, quadratic weighted kappa, and the intraclass correlation coefficient (ICC) were calculated for the raw final VCP/p97 scores.

An exploratory subgroup survival analysis restricted to patients with FIGO stage I–II disease was also performed, in order to assess whether VCP/p97 expression was associated with overall survival within early-stage disease. Due to the limited number of events in this subgroup, the results should be interpreted cautiously and considered as hypothesis-generating.

A two-sided *p*-value was used in all statistical analyses, and *p* < 0.05 was considered statistically significant.

## 3. Results

### 3.1. Clinicopathological Features of the Patients

A total of 85 patients diagnosed with EEC were included in the study. The mean age of the patients was 61.1 ± 10.6 years, with a median age of 62 years, ranging from 31 to 81 years. The mean tumor diameter was 3.9 ± 1.9 cm. When the FIGO grade distribution was evaluated, 38 cases (44.7%) were grade I, 33 cases (38.8%) were grade II, and 14 cases (16.5%) were grade III tumors. With respect to the depth of myometrial invasion, it was <50% in 22 cases (25.9%) and ≥50% in 63 cases (74.1%). Lymphovascular invasion was present in 19 cases (22.4%) and absent in 66 cases (77.6%). Cervical invasion was detected in 16 cases (18.8%), whereas 69 cases (81.2%) had no cervical invasion. Regarding adnexal involvement, 10 cases (11.8%) showed adnexal involvement, while 75 cases (88.2%) did not. Lymph node metastasis was present in 16 cases (18.8%), whereas no lymph node metastasis was detected in 69 cases (81.2%). Distant metastasis was present at diagnosis/surgical staging in 11 cases (12.9%), whereas 74 cases (87.1%) presented no distant metastasis. According to the 2023 FIGO staging system, 45 cases (52.9%) were classified as stage I, 14 cases (16.5%) as stage II, 15 cases (17.6%) as stage III, and 11 cases (12.9%) as stage IV. In the grouped FIGO stage analysis, 59 cases (69.4%) were stage I–II and 26 cases (30.6%) were stage III–IV. According to the immunohistochemical evaluation of VCP/p97 expression, 49 cases (57.6%) were included in the low VCP/p97 expression group, whereas 36 cases (42.4%) were included in the high VCP/p97 expression group. The distribution of final VCP/p97 expression scores among the cases was as follows: a score of 1 in 2 cases, a score of 2 in 10 cases, a score of 3 in 2 cases, a score of 4 in 35 cases, a score of 6 in 31 cases, and a score of 9 in 5 cases.

### 3.2. VCP/p97 Expression and Its Association with Clinicopathological Features

The relationship between the clinicopathological characteristics of the cases and VCP/p97 expression status is presented in Table 1.

The mean age was 58.9 ± 10.8 years in the low VCP/p97 expression group and 64.0 ± 9.7 years in the high VCP/p97 expression group. Patients with high VCP/p97 expression had a significantly higher mean age than those in the low-expression group (*p* = 0.025). The mean tumor diameter was 3.1 ± 1.5 cm in the low VCP/p97 expression group and 4.9 ± 1.9 cm in the high VCP/p97 expression group. Tumor diameter was significantly larger in the high VCP/p97 expression group (*p* < 0.001).

With respect to the FIGO grade distribution, 45 cases (91.8%) were grade I–II and 4 cases (8.2%) were grade III in the low VCP/p97 expression group, whereas 26 cases (72.2%) were grade I–II and 10 cases (27.8%) were grade III in the high VCP/p97 expression group. The proportion of grade III cases was significantly higher in the high VCP/p97 expression group (*p* = 0.016).

When the depth of myometrial invasion was evaluated, 19 cases (38.8%) in the low VCP/p97 expression group had <50% myometrial invasion, whereas 30 cases (61.2%) had ≥50% myometrial invasion. In contrast, in the high VCP/p97 expression group, only 3 cases (8.3%) had <50% myometrial invasion, while 33 cases (91.7%) had ≥50% myometrial invasion. As such, deep myometrial invasion was significantly more frequent in the high VCP/p97 expression group (*p* = 0.002).

Lymphovascular invasion was detected in 3 cases (6.1%) in the low VCP/p97 expression group and in 16 cases (44.4%) in the high VCP/p97 expression group. The presence of lymphovascular invasion was significantly associated with high VCP/p97 expression (*p* < 0.001).

Cervical invasion was present in 3 cases (6.1%) in the low VCP/p97 expression group and in 13 cases (36.1%) in the high VCP/p97 expression group (*p* < 0.001). Adnexal involvement was observed in only 1 case (2%) in the low VCP/p97 expression group, whereas it was detected in 9 cases (25.0%) in the high VCP/p97 expression group (*p* = 0.002).

Lymph node metastasis was observed in 3 cases (6.1%) in the low VCP/p97 expression group and in 13 cases (36.1%) in the high VCP/p97 expression group. Lymph node metastasis was significantly associated with high VCP/p97 expression (*p* < 0.001). Distant metastasis was not observed in the low VCP/p97 expression group, whereas it was detected in 11 cases (30.6%) in the high VCP/p97 expression group (*p* < 0.001).

With respect to FIGO stage, 45 cases (91.8%) were stage I–II and 4 cases (8.2%) were stage III–IV in the low VCP/p97 expression group, whereas 14 cases (38.9%) were stage I–II and 22 cases (61.1%) were stage III–IV in the high VCP/p97 expression group. Advanced FIGO stage was significantly more frequent in the high VCP/p97 expression group (*p* < 0.001).

Interobserver agreement for VCP/p97 immunohistochemical scoring was excellent. For the dichotomized low/high VCP/p97 expression classification, the unweighted Cohen’s κ was 1.000. For the raw final VCP/p97 scores, the linear weighted κ was 0.966, the quadratic weighted κ was 0.986, and the ICC was 0.993. These analyses were based on the independent scores assigned by the two observers before consensus review.

### 3.3. Survival Analysis

The mean follow-up duration of the cohort was 71.5 ± 40.3 months, the median follow-up duration was 68 months, and the follow-up period ranged from 2 to 174 months. During the follow-up period, 26 cases (30.6%) died, while 59 cases (69.4%) were considered alive or censored. In the low VCP/p97 expression group, death occurred in 3 of 49 cases (6.1%) whereas, in the high VCP/p97 expression group, death was observed in 23 of 36 cases (63.9%).

**Figure 2:**
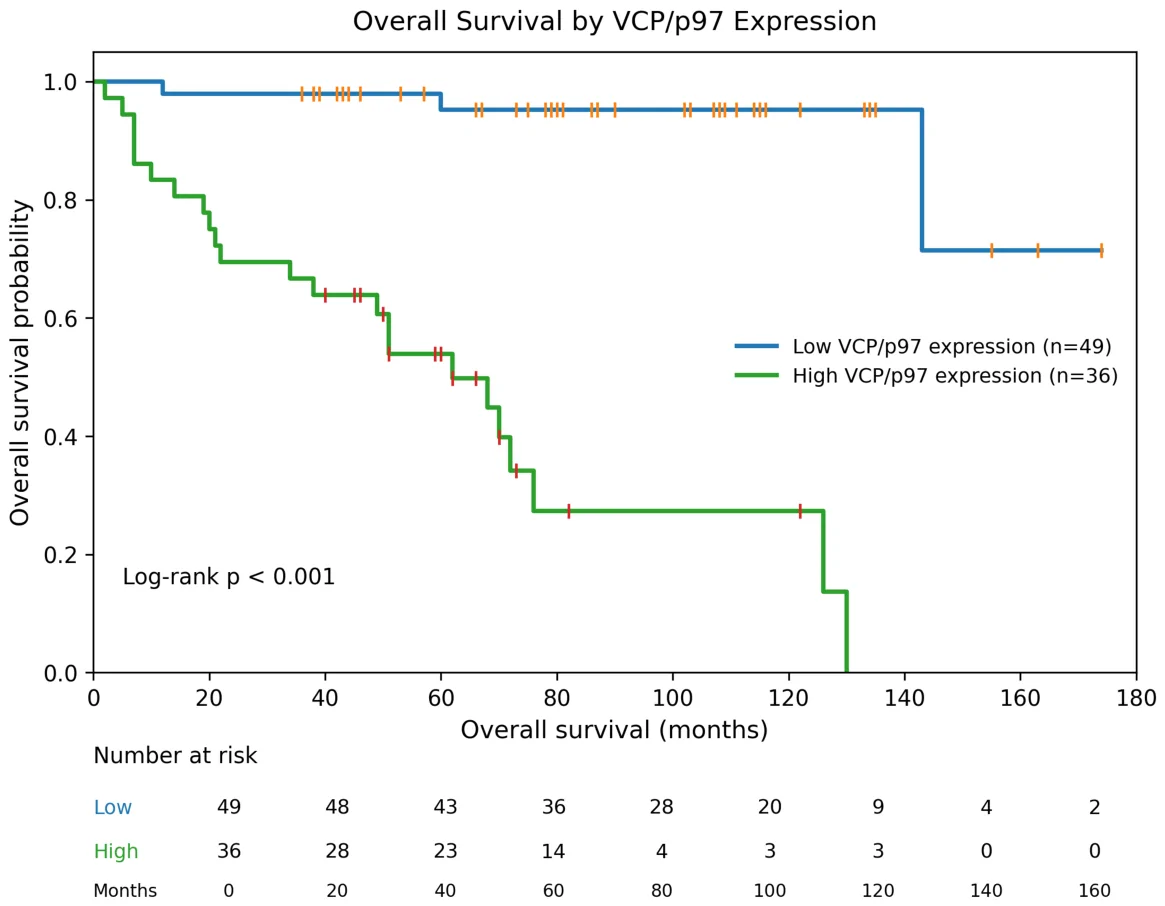
Kaplan–Meier overall survival analysis according to VCP/p97 expression status in patients with endometrioid endometrial carcinoma. Patients with high VCP/p97 expression showed significantly poorer overall survival compared with those with low VCP/p97 expression (log-rank *p* < 0.001). The blue line represents the low VCP/p97 expression group (*n* = 49), and the green line represents the high VCP/p97 expression group (*n* = 36). Censored cases are indicated by tick marks on the survival curves. Numbers at risk are displayed below the Kaplan–Meier plot.

The Kaplan–Meier analysis results demonstrated a marked difference in overall survival curves according to VCP/p97 expression status. Overall survival was significantly lower in patients with high VCP/p97 expression than in those with low expression (log-rank *p* < 0.001; Figure 2). The median overall survival was calculated as 62 months in the high VCP/p97 expression group, whereas the median overall survival was not reached in the low VCP/p97 expression group because the survival curve did not fall below the 50% survival level during the follow-up period. Numbers at risk are displayed below the Kaplan–Meier plot to show the number of patients remaining under observation in each VCP/p97 expression group at predefined follow-up time points.

### 3.4. Univariate and Multivariate Analyses

Univariate and multivariate Cox regression analyses were performed to evaluate factors affecting overall survival (Table 2). In the univariate Cox regression analysis, age, FIGO grade III, advanced FIGO stage, and high VCP/p97 expression were significantly associated with overall survival. In particular, increasing age was associated with poorer overall survival (HR: 1.05; 95% CI: 1.01–1.10; *p* = 0.007); FIGO grade III tumors were associated with poorer overall survival when compared with grade I–II tumors (HR: 3.54; 95% CI: 1.50–8.39; *p* = 0.004); advanced FIGO stage was associated with a markedly increased risk of death when compared with stage I–II disease (HR: 13.31; 95% CI: 4.81–36.84; *p* < 0.001); and high VCP/p97 expression showed a strong association with poorer overall survival when compared with low expression (HR: 28.57; 95% CI: 6.61–123.46; *p* < 0.001).

In the multivariate Cox regression model, VCP/p97 expression, age, FIGO grade, and FIGO stage were evaluated together. In the multivariate analysis, high VCP/p97 expression remained significantly associated with poorer overall survival after adjustment for other variables (HR: 8.99; 95% CI: 1.77–45.65; *p* = 0.008). Age also remained significantly associated with overall survival in the multivariate model (HR: 1.05; 95% CI: 1.00–1.10; *p* = 0.034), and advanced FIGO stage was independently associated with poorer overall survival (HR: 5.47; 95% CI: 1.74–17.15; *p* = 0.004). In contrast, although FIGO grade III was significant in the univariate analysis, it did not retain statistical significance in the multivariate model (HR: 2.12; 95% CI: 0.86–5.23; *p* = 0.104).

The proportional hazards assumption was assessed for the multivariate Cox regression model. No statistically significant violation of the proportional hazards assumption was detected for VCP/p97 expression (*p* = 0.419), age (*p* = 0.057), FIGO grade (*p* = 0.716), or FIGO stage (*p* = 0.120). The global test was also not statistically significant (*p* = 0.115), indicating no clear evidence against the proportional hazards assumption for the multivariate model.

### 3.5. Cut-Off Sensitivity Analyses for VCP/p97 Expression

Cut-off sensitivity analyses were performed using alternative final score thresholds for high VCP/p97 expression. At cut-offs of >2 and >3, all 26 deaths occurred in the high-expression groups (log-rank *p* = 0.019 and *p* = 0.012, respectively), resulting in complete separation and precluding Cox hazard ratio estimation. At a cut-off of >4, 3 deaths occurred among 49 low-expression cases and 23 among 36 high-expression cases (log-rank *p* < 0.001; HR, 28.57; 95% CI, 6.61–123.46). At a cut-off of >6, 21 of 80 low-expression and all 5 high-expression cases died (log-rank *p* < 0.001), although it should be noted that Cox estimation was limited by the small high-expression group (Table 3). Overall, the sensitivity analyses showed that higher VCP/p97 expression was consistently associated with poorer overall survival across the explored thresholds. However, as the cut-offs were evaluated in a retrospective cohort and some thresholds were affected by sparse events, complete separation, or very small subgroup size, these findings should be interpreted as exploratory.

### 3.6. Exploratory FIGO Stage I–II Subgroup Survival Analysis

An exploratory subgroup survival analysis was performed in patients with FIGO stage I–II disease. Among the 59 patients with stage I–II EEC, 45 were in the low VCP/p97 expression group and 14 were in the high VCP/p97 expression group. Death occurred in 2 patients in the low-expression group and 6 patients in the high-expression group.

**Figure 3:**
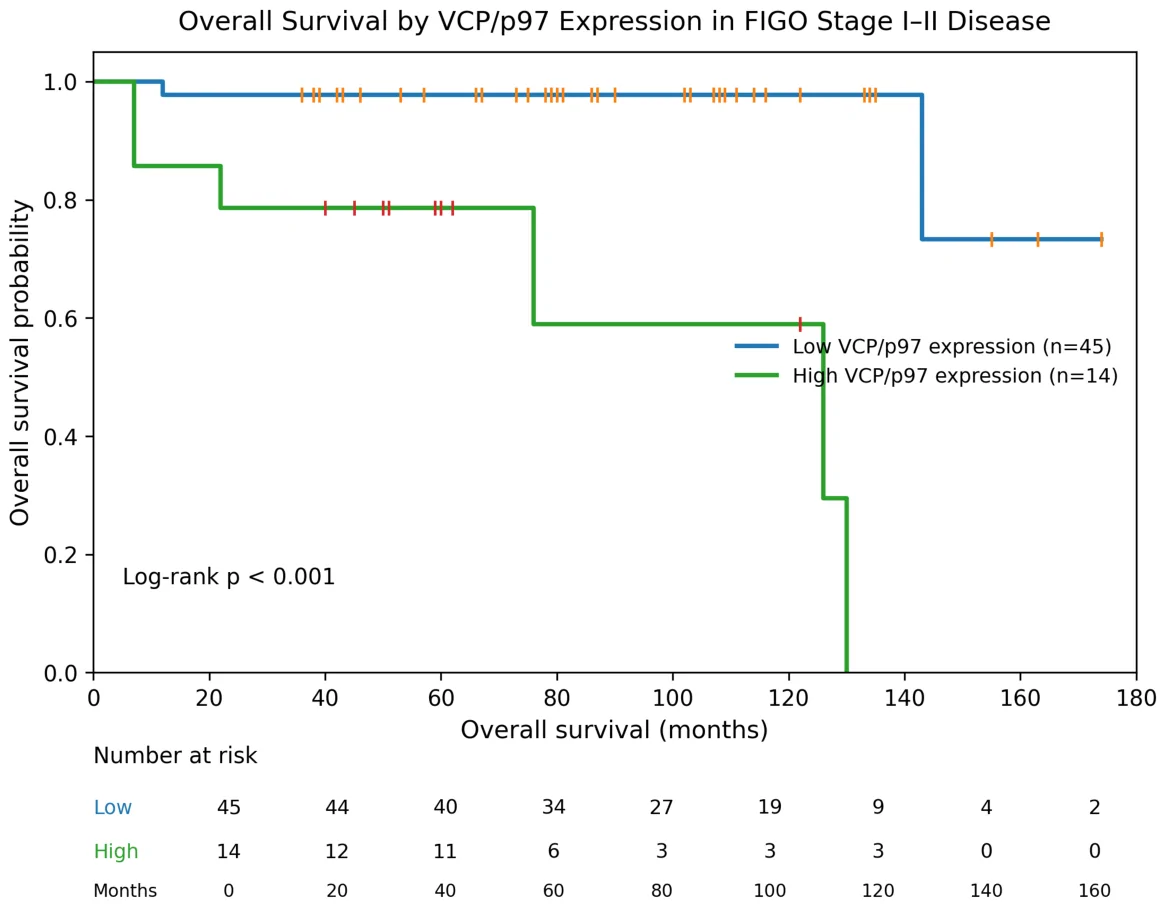
Kaplan–Meier overall survival analysis according to VCP/p97 expression status in patients with FIGO stage I–II endometrioid endometrial carcinoma. High VCP/p97 expression was associated with poorer overall survival compared with low VCP/p97 expression in this exploratory subgroup analysis (log-rank *p* < 0.001). The blue and green lines represent the low-expression (*n* = 45) and high-expression (*n* = 14) groups, respectively. Tick marks indicate censored cases, and numbers at risk are shown below the plot.

The Kaplan–Meier analysis results showed significantly poorer overall survival in the high VCP/p97 expression group within the FIGO stage I–II subgroup, according to the log-rank test (*p* < 0.001). In the univariate Cox regression analysis, high VCP/p97 expression was associated with poorer overall survival in this subgroup (HR: 27.94; 95% CI: 3.30–236.77; *p* = 0.002; Figure 3).

As only 8 deaths occurred in the FIGO stage I–II subgroup and the confidence interval was wide, this analysis was considered exploratory and hypothesis-generating. Therefore, no multivariate Cox regression analysis was performed for this subgroup.

## 4. Discussion

In this study, the prognostic significance of immunohistochemical VCP/p97 expression was investigated in terms of clinicopathological parameters and overall survival in EEC patients. High VCP/p97 expression was significantly associated with adverse clinicopathological parameters—namely, older age, larger tumor diameter, high FIGO grade, deep myometrial invasion, lymphovascular or cervical invasion, adnexal involvement, lymph node metastasis, distant metastasis, and advanced FIGO stage—in EEC cases. In addition, overall survival was found to be markedly worse in patients with high VCP/p97 expression when compared with the low-expression group. Many of these parameters are anatomically and statistically related, and may represent different components of the same advanced disease phenotype. Therefore, these findings suggest that high VCP/p97 expression may be associated with a more aggressive/advanced pathological phenotype in EEC.

Prognostic evaluation in endometrial cancer is traditionally based on clinicopathological parameters such as the histological type, depth of myometrial invasion, lymphovascular invasion, cervical stromal invasion, lymph node metastasis, distant metastasis, and FIGO stage and grade. The current FIGO approach emphasizes the need to evaluate histopathological parameters, surgical stage, lymphovascular invasion, and molecular classification collectively for risk stratification and treatment planning [5,7]. Although molecular classification informs the prognostic evaluation in endometrial cancer, the current data indicate that surgical stage and tumor extension remain valid predictors at the clinical level [8]. Despite the fact that VCP/p97 expression was found to be associated with anatomical dissemination and survival in this series, which may have potential clinicopathological importance in this context, confirmation of this relationship in larger series with known molecular profiles will be required to shed light on the subject.

VCP/p97 is an important component of the ubiquitin–proteasome system and the ERAD pathway, as it enables ubiquitinated substrates to be separated from protein complexes or cellular structures and directed toward degradation pathways [12]. It also plays a role in processes such as autophagy, aggresome clearance, DNA damage response, cell cycle, and apoptosis [14]. Cancer cells may be highly dependent on proteostasis mechanisms due to increased protein synthesis, genomic instability, and metabolic stress. Therefore, VCP/p97 overexpression may reflect an adaptive mechanism that contributes to the survival of tumor cells under proteotoxic stress. VCP/p97 has also been shown to function at different steps of autophagic flux processes within the protein quality control system and to play a critical role in aggresome clearance [16,17]. Tumor cells, particularly in rapidly growing areas exposed to a hypoxic microenvironment, may utilize proteostasis and autophagy pathways to prevent the accumulation of misfolded proteins. Therefore, increased VCP/p97 expression may be an adaptation that allows tumor cells to survive under stress.

The association between high VCP/p97 expression and local invasion or metastatic spread observed in our cohort is biologically plausible, although it remains inferential. Experimental studies have suggested that VCP/p97 may regulate actin organization and cell motility through the RhoA–ROCK pathway, and may influence cellular processes involving endoplasmic reticulum stress, epithelial–mesenchymal transition, migration, invasion, and cell death [18,19]. These experimental findings provide a potential biological framework for interpreting the association between high VCP/p97 expression and invasive or metastatic tumor features observed in our cohort. Nevertheless, given the retrospective and immunohistochemical nature of our study, these findings should be interpreted as associative and do not establish a direct causal role of VCP/p97 in promoting invasion or metastasis in EEC.

The findings of this study are generally consistent with previous studies reporting that VCP/p97 plays an adverse prognostic role in solid tumors. In the review by Costantini et al., high VCP/p97 expression was reported to be associated with invasion, metastasis, recurrence, chemotherapy resistance, and shorter survival in gastrointestinal, lung, breast, prostate, head and neck, thyroid, hematological, and some gynecological malignancies [14]. In colorectal carcinoma, high VCP/p97 expression has been found to be associated with advanced invasion depth, venous invasion, advanced stage, high recurrence, and poor survival [21]. In gastric carcinoma, high VCP/p97 expression was found to be associated with greater tumor size, vascular and lymphatic invasion, lymph node metastasis, and shorter disease-free/overall survival [22]. In hepatocellular carcinoma, VCP/p97 has been shown to have prognostic value for disease-free and overall survival and to affect cell growth and migration in experimental models [23]. In a recent study conducted in clear cell renal cell carcinoma, high VCP/p97 expression was found to be associated with large tumor size, high Fuhrman nuclear grade, advanced TNM stage, and distant metastasis. Survival was found to be significantly worse in the high expression group, and VCP/p97 was indicated as an adverse prognostic factor [37]. These data support the possibility that high VCP/p97 expression may be related to more aggressive tumor biology across a range of different cancer types.

Nevertheless, the prognostic role of VCP/p97 does not appear to be uniform in all malignancies; for example, Meyer et al. reported no significant association between VCP/p97 expression and overall survival in an oropharyngeal squamous cell carcinoma cohort, and higher VCP/p97 expression was associated with better disease-free survival in HPV-negative cases [38]. This discordant finding is important because it suggests that the biological and prognostic relevance of VCP/p97 may be tumor-type dependent and may be influenced by the molecular background, viral status, tumor microenvironment, and differences in immunohistochemical scoring methods. Therefore, the association observed in the present EEC cohort should be interpreted cautiously and validated independently.

There are studies in the endometrial cancer literature supporting the prognostic value of immunohistochemical biomarkers. It has been reported that erythropoietin expression in endometrial carcinoma may be associated with a worse clinical outcome and may be an independent prognostic factor [39]. An assessment of the associations between NGAL and VEGF expression levels and FIGO stage, differentiation level, and myometrial invasion demonstrated that protein markers reflecting tumor progression in other contexts may also have value in endometrial cancer [40]. In a large retrospective analysis examining mesothelin and CA125, mesothelin expression and mesothelin/CA125 co-expression were found to be associated with deep myometrial invasion, lymphovascular stromal invasion, poor progression-free survival, and poor overall survival; subsequently, they were indicated as adverse prognostic factors in a multivariate analysis [41]. These examples demonstrate that immunohistochemistry-based protein expression analyses may have diagnostic and prognostic value in the context of endometrial cancer, with the observed association between VCP/p97 expression and overall survival in EEC further contributing to this line of research. In addition to molecular classification, approaches focused on the use of immunohistochemical biomarkers may also have value in endometrial cancer, especially as clinical heterogeneity persists even within molecular subgroups. In this regard, studies showing that hormone receptors maintain their prognostic value within molecular subgroups have demonstrated that immunohistochemical biomarkers may provide complementary contributions when used in addition to molecular classification [10]. Therefore, studies evaluating VCP/p97 together with molecular subtypes should be conducted in the future.

Studies on VCP/p97 in experimental models of the female genital system have been reported in the literature. For example, in choriocarcinoma cells, VCP/p97 inhibition or siRNA-mediated suppression was found to be associated with autophagic protein accumulation, G0/G1 cell cycle arrest, accumulation of ubiquitinated proteins, and caspase activation [33]. In uterine leiomyosarcoma models, proteotoxic stress was shown to induce a more pronounced apoptotic response in cancer cells compared with normal uterine smooth muscle cells, and the proteostasis response was shown to affect tumor cell sensitivity [42]. In ovarian cancer, VCP inhibitors have been reported to induce cell death through endoplasmic reticulum stress and unfolded protein response [32]. Although these studies were not conducted directly in endometrial tissues, they provide indirect evidence supporting the biological importance of VCP/p97 in gynecological or related tumor models.

Although clinical data on VCP/p97 specifically in endometrial cancer remain limited, experimental studies have indicated that the ubiquitin–proteasome system and related protein degradation pathways may play roles in endometrial tumor biology. UCHL5 has been reported to accelerate tumor growth by activating the Wnt/β-catenin signaling pathway in endometrial cancer [35]. Similarly, the roles of the ubiquitin–proteasome system and proteasome-targeted approaches in cancer therapy support the importance of these pathways in the context of tumor biology [15]. In endometrial cancer, the combination of disulfiram and copper-cysteamine has been reported to exert antitumor effects through mitochondrial damage, suppression of DNA repair pathways, and apoptosis; in particular, disulfiram–copper complexes were stated to affect the p97–NPL4–UFD1 pathway through the p97 adaptor NPL4 [36]. Evaluating these experimental data together with our clinical findings, it is reasonable to hypothesize that VCP/p97-related proteostasis mechanisms have biological and potential therapeutic importance in endometrial cancer.

The survival-related findings of the present study should also be interpreted carefully. High VCP/p97 expression remained associated with poorer overall survival in the multivariate Cox regression model after adjustment for age, FIGO grade, and FIGO stage. In addition, the exploratory analysis restricted to FIGO stage I–II disease suggested that VCP/p97 expression may retain prognostic relevance within early-stage tumors; however, the number of deaths in this subgroup was small and the confidence interval was wide. Similarly, the cut-off sensitivity analyses showed that higher VCP/p97 expression was consistently associated with poorer survival across explored thresholds, but some thresholds were limited by sparse events, complete separation, or a very small subgroup size. Therefore, the survival analyses should be regarded as exploratory and hypothesis-generating, and the selected cut-off should be interpreted with awareness of potential optimism bias.

The therapeutic targetability of VCP/p97 has also been investigated in different tumors. In acute myeloid leukemia models, p97 inhibition has been reported to exert antitumor effects, which were associated with the accumulation of ubiquitinated proteins, activation of the unfolded protein response, and apoptosis [34]. In cholangiocarcinoma, VCP has been shown to support tumor development by increasing ubiquitin-dependent degradation of BAP1; conversely, VCP inhibitors were shown to suppress tumor cell growth by preventing BAP1 degradation [43]. Studies on VCP/p97 inhibitors have shown that, in some tumors, this protein may serve not only as a prognostic marker but also a targetable molecular node. Although the treatment response was not directly examined in the present study, the finding that high VCP/p97 expression is associated with poor survival suggests that this protein may be a candidate for further research regarding endometrial cancer therapies.

This study has several limitations. First, it was retrospective and single-center in design, and the final cohort included only 85 surgically nodal-assessed patients. This selection may have enriched the cohort in patients with higher-risk features, as nodal assessment is more commonly performed in patients with clinically or pathologically increased risk. Second, the number of survival events was limited, particularly in the low VCP/p97 expression group, resulting in wide confidence intervals in the survival models. Third, recurrence data, disease-free survival, adjuvant treatment information, comorbidities, and cause-specific mortality were not available; therefore, overall survival may have been influenced by non-cancer-related deaths. Fourth, molecular classification data—including POLE mutation status, MMR protein expression, p53 status, and ER/PR expression—were not available. Therefore, we could not determine whether VCP/p97 expression provides prognostic information independent of TCGA-based molecular subgroups. Finally, the immunohistochemical cut-off used in this study requires further external validation.

## 5. Conclusions

High immunohistochemical VCP/p97 expression was found to be associated with adverse clinicopathological features and poorer overall survival in patients with EEC. These findings suggest that higher VCP/p97 may be related to a more aggressive or advanced pathological phenotype and, thus, VCP/p97 may represent a candidate tissue-based marker associated with tumor progression features. Larger multicenter studies incorporating recurrence data, disease-specific survival, adjuvant treatment information, comorbidity data, and molecular classification are needed to clarify the clinical significance of VCP/p97 expression in the context of EEC.

## Figures and Tables

**Table 1:** Clinicopathological characteristics according to VCP/p97 expression status.

Variable	Total Cohort (*n* = 85)	Low VCP/p97 Expression (*n* = 49)	High VCP/p97 Expression (*n* = 36)	*p*-Value
Age, years, mean ± SD	61.1 ± 10.6	58.9 ± 10.8	64.0 ± 9.7	0.025
Tumor size, cm, mean ± SD	3.9 ± 1.9	3.1 ± 1.5	4.9 ± 1.9	<0.001
FIGO grade, *n* (%)				0.016
I–II	71 (83.5)	45 (91.8)	26 (72.2)	
III	14 (16.5)	4 (8.2)	10 (27.8)	
Myometrial invasion, *n* (%)				0.002
<50%	22 (25.9)	19 (38.8)	3 (8.3)	
≥50%	63 (74.1)	30 (61.2)	33 (91.7)	
Lymphovascular invasion, *n* (%)				<0.001
Absent	66 (77.6)	46 (93.9)	20 (55.6)	
Present	19 (22.4)	3 (6.1)	16 (44.4)	
Cervical invasion, *n* (%)				<0.001
Absent	69 (81.2)	46 (93.9)	23 (63.9)	
Present	16 (18.8)	3 (6.1)	13 (36.1)	
Adnexal involvement, *n* (%)				0.002
Absent	75 (88.2)	48 (98.0)	27 (75.0)	
Present	10 (11.8)	1 (2.0)	9 (25.0)	
Lymph node metastasis, *n* (%)				<0.001
Absent	69 (81.2)	46 (93.9)	23 (63.9)	
Present	16 (18.8)	3 (6.1)	13 (36.1)	
Distant metastasis, *n* (%)				<0.001
Absent	74 (87.1)	49 (100.0)	25 (69.4)	
Present	11 (12.9)	0 (0.0)	11 (30.6)	
FIGO stage, *n* (%)				<0.001
I–II	59 (69.4)	45 (91.8)	14 (38.9)	
III–IV	26 (30.6)	4 (8.2)	22 (61.1)	

Data are presented as mean ± SD or *n* (%), as appropriate. Age and tumor size were compared using Student’s *t*-test. Categorical variables were compared using the chi-square test or Fisher’s exact test, as appropriate. FIGO grade and FIGO stage were dichotomized for statistical comparisons. Two-sided *p*-values < 0.05 were considered statistically significant. SD, standard deviation; FIGO, International Federation of Gynecology and Obstetrics; VCP/p97, valosin-containing protein/p97.

**Table 2:** Univariate and multivariate Cox proportional hazards regression analyses for overall survival.

Variables	Univariate Analysis		Multivariate Analysis	
	HR (95% CI)	*p*-Value	HR (95% CI)	*p*-Value
Age (years)	1.05 (1.01–1.10)	0.007	1.05 (1.00–1.10)	0.034
FIGO grade				
III vs. I–II	3.54 (1.50–8.39)	0.004	2.12 (0.86–5.23)	0.104
FIGO stage				
III–IV vs. I–II	13.31 (4.81–36.84)	<0.001	5.47 (1.74–17.15)	0.004
VCP/p97 expression				
High vs. low	28.57 (6.61–123.46)	<0.001	8.99 (1.77–45.65)	0.008

HR, hazard ratio; CI, confidence interval; FIGO, International Federation of Gynecology and Obstetrics; VCP/p97, valosin-containing protein/p97. Variables entered into the multivariate Cox proportional hazards regression model included VCP/p97 expression, age, FIGO grade, and FIGO stage. Lymph node metastasis was not included in the multivariate model because it is incorporated into FIGO stage and is closely related to advanced-stage disease. Hazard ratios are presented with corresponding 95% confidence intervals. Two-sided *p*-values < 0.05 were considered statistically significant.

**Table 3:** Cut-off sensitivity analyses for VCP/p97 expression and overall survival.

Cut-Off for High	Low-Expression	High-Expression	Log-Rank	Univariate Cox
VCP/p97 Expression	Group, *n*/Events	Group, *n*/Events	*p*-Value	HR (95% CI)
>2	12/0	73/26	0.019	Not estimable
>3	14/0	71/26	0.012	Not estimable
>4	49/3	36/23	<0.001	28.57 (6.61–123.46)
>6	80/21	5/5	<0.001	Not estimable

HR, hazard ratio; CI, confidence interval. Events refer to deaths during follow-up. Hazard ratios were reported as not estimable when complete separation, sparse events, or very small subgroup size precluded reliable Cox regression estimation.

## Data Availability

The original contributions presented in this study are included in the article. Further inquiries can be directed to the corresponding author.

## Abbreviations

The following abbreviations are used in this manuscript:

AAA+	ATPases associated with diverse cellular activities
BAP1	BRCA1-associated protein 1
CI	Confidence interval
EEC	Endometrioid endometrial carcinoma
ERAD	Endoplasmic reticulum-associated degradation
FIGO	International Federation of Gynecology and Obstetrics
HR	Hazard ratio
MMR	Mismatch repair
NGAL	Neutrophil gelatinase-associated lipocalin
NPL4	Nuclear protein localization protein 4
OS	Overall survival
POLE	DNA polymerase epsilon
SD	Standard deviation
siRNA	Small interfering RNA
TCGA	The Cancer Genome Atlas
UCHL5	Ubiquitin C-terminal hydrolase L5
UFD1	Ubiquitin recognition factor in ER-associated degradation protein 1
VCP/p97	Valosin-containing protein/p97
VEGF	Vascular endothelial growth factor
